# A dual inhibitor of TrxR1 and XIAP induces pyroptosis in melanoma

**DOI:** 10.3389/fcell.2025.1542356

**Published:** 2025-05-23

**Authors:** Yuan Wang, Xiangmei Li, Xinyue Dong, Haokun Yuan, Ruiqin Fang, Ran Zhang, Wei-jia Wang

**Affiliations:** ^1^ The School of Medicine, University of Electronic Science and Technology of China, Chengdu, China; ^2^ The Key Laboratory for Human Disease Gene Study of Sichuan Province and the Department of Laboratory Medicine, Sichuan Provincial People’s Hospital, School of Medicine, University of Electronic Science and Technology of China, Chengdu, China; ^3^ Division of Neonatology, Department of Pediatrics, The Affiliated Hospital of Southwest Medical University, Luzhou, China; ^4^ The School of Life Science, University of Electronic Science and Technology of China, Chengdu, China; ^5^ Faculty of Science and Engineering, University of Groningen, Groningen, Netherlands; ^6^ Fujian Provincial Key Laboratory of Translational Cancer Medicine, Clinical Oncology School of Fujian Medical University, Fujian Cancer Hospital, Fuzhou, China; ^7^ State Key Laboratory of Cellular Stress Biology, School of Life Sciences, Xiamen University, Xiamen, China

**Keywords:** melanoma, pyroptosis, thioredoxin reductase 1 (TrxR1), X-linked inhibitor of apoptosis (XIAP), reactive oxygen species

## Abstract

Malignant melanoma ranks among the most aggressive forms of cancer, with high rates of metastasis and recurrence, as well as a poor prognosis. Consequently, an urgent need is to develop novel precision therapeutic strategies and corresponding drugs. Previous studies have shown that both X-linked inhibitor of apoptosis (XIAP) and thioredoxin reductase 1 (TrxR1) participate in the resistance of melanoma to chemotherapy-induced cell death. In this study, we designed and synthesized a series of derivatives of natural compounds derived from *Toona sinensis* to simultaneously inhibit TrxR1 activity and destabilize the XIAP protein. The new dual-target inhibitor TRI-03 has significant antiproliferative effects on melanoma cells. Mechanistically, TRI-03 not only increases intracellular reactive oxygen species (ROS) levels by inhibiting TrxR1 activity but also decreases XIAP expression, leading to the activation of the caspase-9/caspase-3/GSDME axis and irreversible GSDME-mediated pyroptosis in melanoma cells. Our *in vivo* animal study confirmed that TRI-03 effectively inhibits melanoma proliferation and metastasis without severe side effects. Therefore, our study identified TRI-03 as a potential antitumor candidate for future development to address melanoma.

## Introduction

Melanoma, a type of skin tumor resulting from the malignant proliferation of melanocytes, has an annual incidence growth rate of 3%–5%, making it one of the fastest-growing malignant tumors worldwide (Sun et al., 2024). Malignant melanoma is also a very aggressive tumor, with high rates of metastasis and recurrence and a poor prognosis (Slominski et al., 2024). Resistance to aggressive treatment regimens is a major challenge in oncology, often because of common genetic and epigenetic alterations that confer resistance to apoptosis in melanoma cells (Lai et al., 2012). Therefore, exploring new precision treatment approaches and identifying more durable and efficient drugs to induce other types of cell death are highly important for advancing melanoma therapy.

The thioredoxin (Trx) system, which consists of NADPH, thioredoxin reductase (TrxR) and Trx, is a critical antioxidant defense mechanism (Gencheva and Arner, 2022). The Trx system maintains the precise balance between the production and removal of reactive oxygen species (ROS) (Lu and Holmgren, 2014). TrxR utilizes its disulfide reductase activity to reduce disulfide bonds (-S-S-) to thiols (-SH), which is crucial for protecting cells from oxidative damage. TrxR reduces Trx, which scavenges harmful ROS, reduces oxidized thiol groups and disulfide bonds in target proteins, and thereby maintains intracellular redox homeostasis (Tinkov et al., 2018).

TrxR1, the most important member of the TrxR family, is aberrantly expressed in several malignancies and is associated with an unfavorable prognosis for patients (Gencheva and Arner, 2022). Recent investigations have identified several TrxR1 inhibitors, particularly metal-containing compounds such as gold(I) NHC complexes, which exert antiproliferative effects on multiple types of cancer cells (Reddy et al., 2018; Karaca et al., 2017; Wang et al., 2024a; Wang et al., 2024b). These gold(I) complexes enter the body, and then their ions undergo redox reactions with sulfur or selenium atoms in reducing molecules, resulting in cytotoxic effects (Lu et al., 2022). TrxR1, with a selenocysteine residue at its catalytic site, is a primary intracellular target for these gold(I) complexes. For example, auranofin, an FDA-approved gold(I) complex for treating rheumatoid arthritis, has attracted attention over the past decade for its cancer-inhibiting properties by directly targeting TrxR1 (Momose et al., 2021). Studies have also documented the effectiveness of auranofin against dacarbazine-resistant acute myeloid leukemia cells, indicating it could be an effective alternative to dacarbazine in cancer therapy (Liu et al., 2020).

XIAP is a key member of the inhibitor of apoptosis protein (IAP) family (Hanifeh and Ataei, 2022). XIAP specifically inhibits caspase-3, caspase-7 and caspase-9, which are critical enzymes in the mitochondrial apoptosis pathway and drive cell death in response to many radiation and chemotherapeutic treatments (Gu et al., 2016). This specificity underscores the role of XIAP in conferring cancer resistance. Elevated XIAP levels are commonly associated with a poor prognosis for patients with various cancers, including melanoma (Tu and Costa, 2020; Daoud et al., 2022). Although some small-molecule antagonists that reduce XIAP expression have been evaluated in preclinical development, they remain clinically unavailable due to limited efficacy or acquired resistance. Therefore, identifying more durable and efficient inhibitors of XIAP, especially through combined dual-target approaches, is crucial for advancing melanoma therapy.

*Toona sinensis*, a traditional Chinese medicine, has recently been found to contain active natural compounds with potential anticancer properties (Yang et al., 2013; Peng et al., 2019). In our preliminary experiments, an alkynyl-containing compound from *T. sinensis* effectively decreased XIAP expression in melanoma cells. Based on this information, we designed and synthesized a series of gold(I) complexes with diphenyl-2-cyclohexylphosphine (R1) and alkyne (R2) units. Among these drugs, TRI-03 exerts the most significant anti-proliferative effects on melanoma cells, outperforming clinical drugs such as dacarbazine and auranofin. Mechanistically, TRI-03 directly inhibits TrxR1 activity, leading to increased intracellular ROS accumulation. TRI-03 also reduces XIAP expression via the autophagy pathway, facilitating caspase activation and resulting in irreversible GSDME-mediated pyroptosis in melanoma cells. Our *in vivo* xenograft study confirmed that TRI-03 exhibits remarkable antitumor activity against melanoma proliferation and metastasis, without severe side effects. In summary, our research identified TRI-03 as a promising lead compound, indicating an innovative pathway for melanoma treatment development.

## Materials and methods

### Cell lines and culture conditions

Melanoma A375 and B16 cells were purchased from Xiamen Immocell Biotechnology Co., Ltd (Xiamen, China). A375 cells were cultured in Dulbecco’s Modified Eagle’s Medium (DMEM) (Thermo Fisher Scientific, Inc., Waltham, United States), while B16 cells were maintained in RPMI-1640 medium (Thermo Fisher Scientific, Inc., Waltham, United States). Both media were supplemented with 10% fetal bovine serum (FBS) (Thermo Fisher Scientific, Inc., Waltham, United States). Regular *mycoplasma* tests confirmed no contamination.

### Antibodies and reagents

The anti-Cleaved Caspase-3 (Cat# 9661S), anti-Caspase-9 (cat #9502), anti-XIAP (cat# 14334) and anti-actin (cat# 4970), goat anti-rabbit (cat# 7074) and goat anti-mouse (cat# 7076) antibodies were purchased from Cell Signaling Technology (Boston, United States). Anti-LC3 (cat# 7543) antibody was purchased from Sigma-Aldrich (St. Louis, United States). Anti-GSDME (Cat# ab215191) antibody was purchased from Abcam (Cambridge, UK). Anti-TrxR1 (cat# 11117-1-AP) and Anti-Trx (cat# 14999-1-AP) antibodies were purchased from Proteintech (Wuhan, China).

Reagents utilized in this study include: Protease inhibitor cocktail (cat# K0010), Phosphatase inhibitor cocktail I (cat# K0021), Phosphatase inhibitor cocktail II (cat# K0022), dacarbazine (cat# HY-10201), 3-methyladenine (3-MA) (cat# HY-19312), MG132 (Cat# HY-13259), Z-VAD-FMK (cat# HY-16658B), Q-VD-OPh (cat# HY-12305), auranofin (cat# A6733), Propidium iodide (PI) (cat# HY-D0815), N-acetylcysteine (NAC) (cat# HY-B0215), necrostatin-1 (Nec-1) (cat# HY-15760), ferrostatin-1 (cat# HY-100579), liproxstatin-1 (cat# HY-12726) and necrosulfonamide (NSA) (cat# HY-100573) were purchased from MedChemExpress (Monmouth Junction, United States); glutathione reductase (GR) (cat# G3664) and recombinant TrxR1 (cat# T9698) were purchased from Sigma Aldrich (St. Louis, United States); and Cycloheximide (Cat# GC17198) was purchased from GlpBio (Pasadena, United States). The U498C TrxR1 mutant (Sec→Cys) was produced following a protocol from previous research (Zhong and Holmgren, 2000).

### Synthesis of gold(I)–phosphine complexes

Using an established protocol, [(PPh_2_Cy)AuCl] was synthesized. First, 0.55 mmol of the alkyne ligand (purchased from Energy Chemical Inc., Shanghai, China) and 0.5 mmol of [(PPh_2_Cy)AuCl] were dissolved in 5 mL of ethanol and stirred for 10 min. Then, 0.6 mmol of KOH dissolved in 0.5 mL of deionized water was added, and the reaction was kept at room temperature for 12 h. Following the reaction, the gold(I)–phosphine complexes were isolated via filtration, followed by recrystallization to yield the final product.

### Cell survival rate

A375 cells were plated in 6-well plates at a density of 4 × 10^5^ cells per well. Following an incubation period of 24 h, the cells were exposed to the prescribed treatments. The cells were subsequently harvested via centrifugation at 2,000 rpm for 5 min and then transferred to 2 mL Eppendorf tubes, after which the supernatant was discarded. After two washes with PBS, the cells were resuspended in 1 mL of PBS buffer containing 5 μg/mL propidium iodide (PI), incubated for 10 min at 4°C in the dark, and subsequently assessed for viability using flow cytometry.

### MTT assay

Briefly, 6 × 10^3^ A375 cells were seeded in 96-well tissue culture plates with 200 μL per well and incubated at 37°C for 72 h. The cells were subsequently exposed to the designated reagents, with DMSO-treated cells serving as the control group. Following the incubation, 10 μL of 5 mg/mL MTT was added to each well, and the plates were incubated at 37°C for 4 h. The culture solution was removed, and DMSO was used to dissolve the stained crystals. After the crystals were completely dissolved, the absorbance was measured at 490 nm. The results were then processed and analyzed using GraphPad Prism.

### Lactate dehydrogenase release assay

LDH release was measured using the CytoTox 96® Non-Radioactive Cytotoxicity Assay Kit according to the manufacturer’s protocol. Specifically, supernatants from both the control and treatment groups were harvested, centrifuged at 3000 r/min for 3 min, and 50 μL from each sample was aliquoted in triplicate into 96-well plates. Each well included 50 μL of 0.5% FBS medium as a background control. Subsequently, 50 μL of the substrate mixture was added to each well and incubated at room temperature for 10 min. The reaction was stopped by the addition of 50 μL of stop solution, and the absorbance was recorded at 490 nm. The percentage of LDH release was determined by adjusting for background values.

### Quantitative real-time PCR

Total RNA was extracted using a Tiangen RNA extraction kit according to the manufacturer’s instructions, dissolved in DEPC water, and then stored at −80°C. For cDNA synthesis, 2 μL of RNA was reverse transcribed using SuperScript® II Reverse Transcriptase (Thermo Fisher Scientific) according to the manufacturer’s protocol. The resulting cDNA was used for quantitative real-time PCR analyses with SYBR Green reagent (Beyotime biotechnology co. ltd), with actin serving as an internal control. The specific primers utilized for real-time PCR are provided below:

TrxR1 (human):

Forward primer: 5′-CATGTCATGTGAGGACGGTCG-3′

Reverse primer: 5′-CTTAGCAGCTGCCAGACCTC-3′

*TrxR2* (human):

Forward primer: 5′-GATTAGGAGGGCGCTTCCG-3′

Reverse primer: 5′-CCTTGGGGAGAAGGTTCCAC-3′

*TrxR3* (human):

Forward primer: 5′-GCAAGCTGCTAGCTCAGAGA-3′

Reverse primer: 5′-TCCACAGCAACCATACTCCAG-3′

*GSDMA* (human):

Forward primer: 5′-CTACAGACCCAGTGAGCCCT-3′

Reverse primer: 5′-CGATGAGGCTGTCAAGTGGT-3′

*GSDMB* (human):

Forward primer: 5′-AACACAAGGGCCAAAGGGAA-3′

Reverse primer: 5′-GGCACTTAGCGAGGGAGTTT-3′

*GSDMC* (human):

Forward primer: 5′-CCTGGTGGTGCCATCCTAAA3′

Reverse primer: 5′-AAGGGAATGCTCCAGGGGTA-3′

*GSDMD* (human):

Forward primer: 5′-CAGTTTCACTTTTAGCTCTGGGC-3′

Reverse primer: 5′-CTGGACCACTCTCCGGACTA-3′

*GSDME* (human):

Forward primer: 5′-GACAGGCCTTGGACTTTCCT-3′

Reverse primer: 5′-AATTCCTGGTTGCTTTGGCA-3′

*Atg7* (human):

Forward primer: 5′-TGTGGTTGCCGGAAGTTGAG-3′

Reverse primer: 5′-GTCCTTGGGAGCTTCATCCA-3′

Atg12 (human):

Forward primer: 5′-AAGTGGGCAGTAGAGCGAAC-3′

Reverse primer: 5′-CACGCCTGAGACTTGCAGTA-3′

### Western blot analysis

The cells were solubilized in 6-well plates with 200 μL of ELB buffer supplemented with protease inhibitors. The lysates were incubated at 4°C for 10 min and then centrifuged at 4°C at 13,000 rpm for 30 min. The protein concentration was determined using the Bradford method in 96-well plates. The samples were subsequently mixed with 2 × SDS loading buffer, heated to boiling, and subjected to SDS‒PAGE for further analysis.

### Cellular TrxR activity assay

A375 cells were seeded into 6-well plates at a density of 4 × 10^5^ cells per well. After a 24-h incubation, the cells were treated with the appropriate drugs for 3 h. Next, the cells were lysed at 4°C in RIPA buffer to obtain total cellular proteins. The protein concentration was determined using the Bradford method in 96-well plates. The cellular TrxR activity in RIPA buffer was subsequently evaluated by the TrxR activity detection kit (Beijing Solarbio Science and Technology Co., Ltd.) following the instructions provided by the manufacturer.

### *In vitro* TrxR activity assays

The DTNB method was employed to measure TrxR activity as previously mentioned (Zhang et al., 2017). In a 96-well plate, wild-type TrxR1 or TrxR1 U498C was incubated with varying concentrations of TRI-03 in TE buffer. Each reaction solution was brought to a final volume of 100 μL and incubated at 37°C for 2 h. Next, 100 μL of a premixed solution containing 2.5 mM DTNB and 200 μM NADPH was added to each well, bringing the final reaction volume to 200 μL. The increase in absorbance at 412 nm over a 3-min period was measured with a microplate reader to verify the linear range of the reaction. TrxR1 activity was determined relative to the control and reported.

### Molecular docking

Protein–ligand molecular docking was conducted by AutoDock as previously described (Morris et al., 2009). The crystal structure of TrxR1 with selenocysteine residue (PDB ID: 3EAN) served as the receptor, and TRI-03 served as the ligand. The receptor and ligand were prepared using MGLTools to generate the corresponding PDBQT files. The docking site was defined by the region including chains A and B of the receptor. Following molecular docking, the receptor-ligand complex with the optimal fit between TrxR1 and TRI-03 was chosen for further analysis by PyMOL and AutoDock.

For the molecular docking of XIAP with pent-1-yn-3-one, a similar process was followed. The complete structure of XIAP was predicted using AlphaFold and used as the receptor, while pent-1-yn-3-one served as the ligand. The subsequent steps were identical to those described above.

### Fluorescence quenching

The fluorescence quenching was previously described (Wang et al., 2015). A 5 μM solution of TrxR protein was combined with varying concentrations of TRI-03 (1–20 μM) in a suitable buffer. The instrument settings on the fluorescence spectrophotometer (Model F-4500) were configured with an excitation wavelength of 280 nm and an emission spectrum recorded from 285 to 430 nm. The slit widths were set to 5 nm for excitation and 2.5 nm for emission, and the measurements were conducted at 25°C. Fluorescence intensities were measured for each TrxR-TRI-03 mixture. The data were plotted as fluorescence intensity against TRI-03 concentration, and *K*_*d*_ values were computed using the Stern–Volmer equation to determine the binding affinity and quenching efficiency.

### Assessment of the Trx redox state

The redox status of Trx was assessed using a previously established protocol (Xu et al., 2022). A375 cells were seeded into 6-well plates at a density of 4 × 10^5^ cells per well. After a 24-h incubation, the cells were treated with the appropriate drugs for 12 h. The cells were subsequently lysed at 4°C in RIPA buffer to obtain total cellular proteins. The protein concentration was determined using the Bradford method in 96-well plates. The samples were subsequently incubated with phenylarsine oxide (PAO) Sepharose for 30 min, and then separated by centrifugation to isolate the reduced and oxidized Trx fractions. Finally, the ratio of oxidized and reduced Trx in the samples was measured using SDS‒PAGE analysis and Western blotting.

### Intracellular GSH/GSSG detection

The levels of intracellular oxidized and reduced glutathione in A375 cells were quantified using a GSH/GSSG Quantification Kit (Nanjing Jiancheng Bioengineering Institute) according to the manufacturer’s guidelines. Briefly, A375 cells were exposed to TRI-03 for 12 h. Following this treatment, the cells were rinsed with PBS and underwent rapid freeze-thaw cycles—alternating between 37°C water and liquid nitrogen. After centrifugation, the supernatants were mixed with the GSH/GSSG detection reagents, followed by recording the absorbance at 415 nm.

### Detection of intracellular ROS levels

The intracellular ROS levels were quantified using CellROX Green reagent following the manufacturer’s protocol. A375 cells were collected and washed twice with PBS, and the CellROX Green reagent was subsequently added to the cell suspension, followed by an incubation at 37°C for 20 min. Upon completion of the incubation period, the supernatant was discarded, and the cells were washed twice with PBS. Green fluorescence was assayed directly using a flow cytometer.

### Lentivirus production

The pCDH lentiviral vector system was used to facilitate the expression of short hairpin RNA (shRNA) in melanoma cells A375. Oligonucleotides designed to target specific genes were subcloned and inserted into the pCDH vector. HEK293T cells were subsequently cotransfected with the pCDH vector and the requisite packaging plasmids to generate lentiviruses. Forty-eight hours after transfection, the viral supernatant was harvested and filtered through a 0.45 μm filter (Millipore, MA, United States) to eliminate the cellular debris. A375 cells were subsequently infected with the lentivirus and incubated for an additional 48 hours. The efficacy of the knockdown was assessed via quantitative real-time PCR. The sequences of the oligonucleotides for the shRNA-targeted mRNAs are provided below:

Non-targeting control (NTC) shRNA, 5′-GCGCGATAGCGCTAATAAT-3′; GSDMA-shRNA, 5′-GGAGATGTGGATGTACCAA-3′; GSDMB-shRNA, 5′- GGACAAGTGGTTAGATGAA-3′; GSDMC-shRNA, 5′- GCATGGTGATGGCTTATAA-3′; GSDMD-shRNA, 5′- GCAGGAGCTTCCACTTCTA-3′; GSDME-shRNA, 5′- GCAGCAAGCAGCTGTTTAT-3′; Atg7-shRNA, 5′-GCTGGTCATCAATGCTGCT-3′; and Atg12-shRNA, 5′- CGAACCATCCAAGGACTCA-3′.

### Animal studies

Specific pathogen-free conditions were maintained for all mice in this study, and sterile food and water were provided, ensuring a controlled environment free from common infectious agents. Compliance with the Institutional Animal Care and Use Committee guidelines at the University of Electronic Science and Technology of China governed the conduct of these animal studies. The protocols were reviewed and approved by the Animal Experimental Ethics Committee (31,679).

### Xenograft formation

A375 cells were subcutaneously injected into the right flanks of thymus-deficient nude mice (BALB/c, male, 7–8 weeks old, 18–20 g) to establish a melanoma xenograft model. The initial tumor volume was approximately 100 mm^3^, confirming the successful establishment of the melanoma xenograft model. Once the tumors were established, the mice were randomly divided into 4 groups (6 mice per group): 1) the vehicle-treated NTC-shRNA group, 2) the TRI-03-treated NTC-shRNA group, 3) the vehicle-treated Atg7-shRNA group, and 4) the TRI-03-treated Atg7-shRNA group. The vehicle and TRI-03 were administered intratumorally to A375 tumor-bearing mice every 2 days. On the 9th day of drug treatment, all the mice were euthanized, and their body weights and tumor weights were recorded. Tumor tissues were then collected for further Western blot analysis.

### The formation of melanoma metastases in the lung

B16 mouse melanoma cells (1 × 10^6^ cells/mouse) were injected into the lateral tail vein of 6-week-old C57BL/6J mice. The mice were then divided into the vehicle, the TRI-03-treated (10 μg per g body weight), and the dacarbazine-treated (10 μg per g body weight) groups. Treatments were administered via a tail vein injection every 2 days. The mice were euthanized at 14 days posttreatment, and the number of lung melanoma metastases was counted. Additionally, renal and liver function parameters were measured.

### Statistical analysis

Most of the data are presented as the means ± SDs derived from replicate experiments or representative repetitions of multiple independent trials, with each experiment involving at least two or three independent determinations. Comparisons among multiple groups were conducted using two-way or one-way ANOVA, followed by Tukey’s or Sidak’s multiple comparison *post hoc* tests. Enzyme activity and cell viability are reported as the mean IC_50_ values ± SEs. Statistical analyses were performed using GraphPad Prism 8 software (La Jolla, United States). Statistical significance is denoted as follows: P < 0.05 for significant differences, P < 0.01 for highly significant differences, P < 0.001 for very highly significant differences, and “ns” for nonsignificant differences.

## Results

### Alkynyl-containing gold(I) complexes exhibit antitumor activity against melanoma cells

**FIGURE 1:**
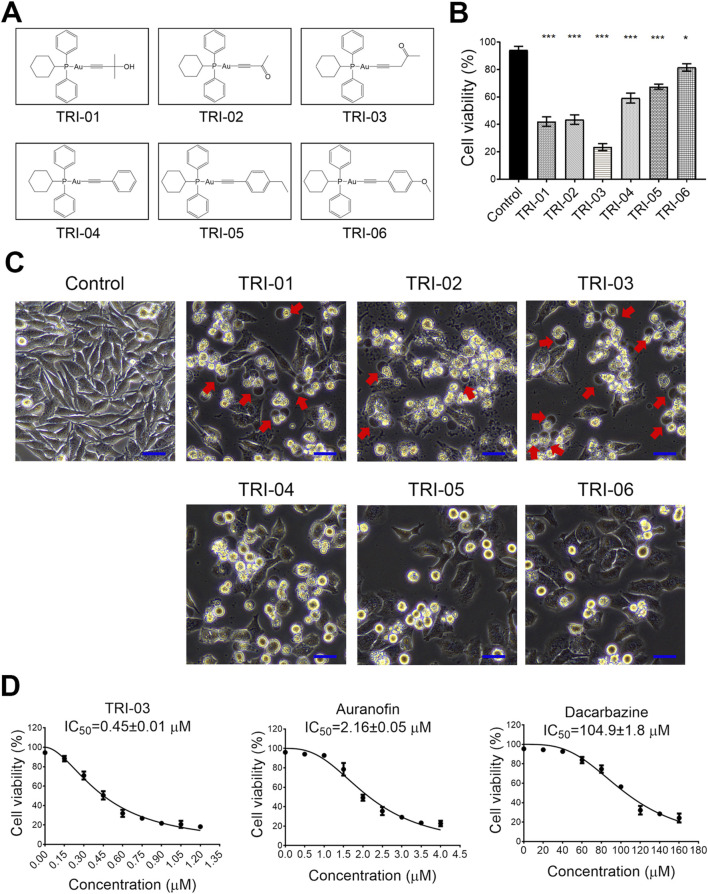
Gold(I) phosphine complexes reveal cytotoxicity to melanoma cells A375. **(A)** The synthesized gold(I) complexes were detailed in their structural form. **(B)** A375 cells were treated with a series of phosphine gold(I) complexes (1 μM, 24 h), and the cell survival was determined by flow cytometry after PI staining. **(C)** The morphological changes in the cells after treatment with various gold(I) complexes (as described in A) were illustrated, with red arrows pointing to the large bubbles originating from the cell membrane. Scale bar, 20 μm (×20 magnification). **(D)** The A375 cells were exposed to various chemicals at the indicated concentrations for 72 h, and their viability was measured using the MTT assay. The IC_50_ values ±SE for TRI-03, auranofin, and dacarbazine were provided. All data, except for that in **(D)**, were presented as the mean ± SD of two or three independent experiments, ***P < 0.001, *P < 0.05.

*Toona sinensis*, a traditional Chinese medicine, has been found to contain natural compounds with potential anticancer properties in recent years (Peng et al., 2019). We analyzed the anticancer mechanisms of these compounds and discovered that pent-1-yn-3-one, an alkynyl-containing compound, effectively reduces XIAP expression in melanoma cells (Supplementary Figures S1A, B). Molecular docking results revealed that it directly binds to the BIR3 domain of XIAP (Supplementary Figure S1C). To further enhance the anticancer efficacy by simultaneously inhibiting both TrxR1 and XIAP, we designed and produced a series of gold(I) complexes featuring alkyne (R2) units (Figure 1A) based on the clinical drug auranofin and its known precursor [(PPh_2_Cy)AuCl].

This synthesis yielded six distinct alkynyl-containing gold(I) complexes (Figure 1A). A375 melanoma cells were treated with these complexes at 1 μM for 24 h to evaluate the antimelanoma potential. Most of the complexes significantly reduced cell viability, with TRI-03 showing the strongest cytotoxic effect (Figure 1B). Notably, TRI-01, TRI-02 and TRI-03 induced the characteristic morphological features of pyroptosis, including cellular swelling and the formation of large bubbles from the cell membrane, whereas the other complexes induced typical apoptotic morphological changes (Figure 1C).

To evaluate the inhibitory effect of TRI-03 on melanoma, A375 cells were exposed to TRI-03 and compared with auranofin and dacarbazine, the latter of which is an FDA-approved therapeutic agent for advanced melanoma. The IC_50_ value of TRI-03 in A375 cells was determined to be 0.45 ± 0.01 μM, which was considerably lower than those of auranofin (2.16 ± 0.05 μM) and dacarbazine (104.9 ± 1.8 μM) (Figure 1D). These findings indicate that alkynyl-containing gold(I) complexes, especially TRI-03, demonstrate strong antitumor activity against melanoma cells.

### TRI-03 induces GSDME-mediated pyroptosis in melanoma cells

**FIGURE 2:**
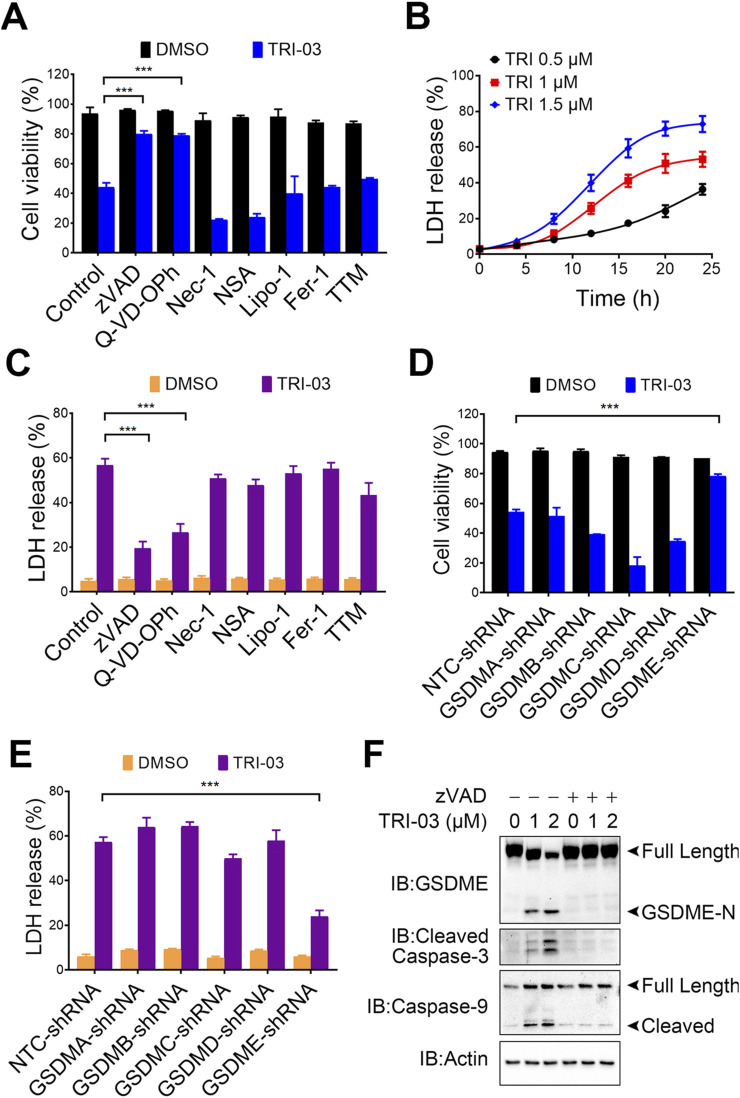
TRI-03 triggers pyroptosis in A375 cells. **(A)** Before incubation with TRI-03 (1 μM, 24 h), A375 cells were pretreated separately for 2 h with various inhibitors: zVAD (20 μM) and Q-VD-OPh (40 μM) for apoptosis or pyroptosis, Nec-1 (20 μM) and NSA (0.5 μM) for necroptosis, Lipo-1 (0.5 μM) and Fer-1 (0.5 μM) for ferroptosis, and TTM (20 μM) for cuproptosis. Cell survival was then assessed using flow cytometry after PI staining. **(B)** A375 cells were exposed to TRI-03 at concentrations of 0.5 μM, 1 μM, or 1.5 μM for the indicated durations, and cell lysis was used for detection of LDH release. **(C)** A375 cells were incubated with each of the inhibitors mentioned in **(A)** for 2 h before TRI-03 (1 μM, 24 h) treatment. Then, the intracellular LDH release was quantified. **(D–E)** In A375 cells, GSDMA, GSDMB, GSDMC, GSDMD, and GSDME were individually knocked down using corresponding shRNAs, and the cells were subsequently treated with TRI-03 (1 μM) for 24 h. Cell survival **(D)** and LDH release **(E)** were then evaluated. **(F)** A375 cells were pretreated with zVAD (20 μM, 2 h), followed by incubation with TRI-03 at the indicated concentrations for another 24 h, and protein levels of GSDME, cleaved caspase-3, and caspase-9 were detected by Western blot. Actin was used as a loading control. All data were presented as the mean ± SD of two or three independent experiments, ***P < 0.001.

To further elucidate the type of cell death induced by TRI-03 in melanoma, A375 cells were pretreated with various cell death inhibitors before TRI-03 administration. Cell viability assays revealed that only the caspase inhibitors (zVAD and Q-VD-OPh), which are known to inhibit apoptosis or pyroptosis, effectively reduced TRI-03-induced cell death (Figure 2A). In contrast, inhibitors of necroptosis (Nec-1 and NSA), ferroptosis (Lipo-1 and Fer-1) and cuproptosis (TTM) had no significant effects (Figure 2A). The release of lactate dehydrogenase (LDH), a marker of cell lysis, also increased in a time-dependent manner following TRI-03 treatment (Figure 2B). Consistent with the cell viability results, LDH release was also inhibited by caspase inhibitors (zVAD and Q-VD-OPh) and was not affected by the other inhibitors examined (Figure 2C).

Since five gasdermin family members (GSDMA, GSDMB, GSDMC, GSDMD, and GSDME) reportedly function as pyroptotic executioners (Zhu et al., 2024), we investigated their involvement in TRI-03-induced pyroptosis. Knockdown of GSDME significantly inhibited TRI-03-induced cell death and LDH release, whereas knockdown of the other gasdermin family members failed to exert this inhibitory effect (Figures 2D,E; Supplementary Figures S1A–E). We performed Western blot analyses to obtain deeper understanding of the pyroptosis pathway induced by TRI-03. TRI-03 treatment dose-dependently induced the cleavage of GSDME, a marker of pyroptosis, as well as cleavage of caspase-3 and caspase-9 (Figure 2F). Caspase-9, an initiator caspase, activates executioner caspase-3, which subsequently cleaves GSDME in a cascade reaction, leading to pyroptic cell death (Wang et al., 2017). Pretreatment with zVAD substantially reduced GSDME cleavage in melanoma cells following TRI-03 administration (Figure 2F), confirming that TRI-03-stimulated GSDME cleavage is dependent on caspase activation.

In conclusion, these results suggest that TRI-03 primarily induces pyroptosis in melanoma cells through the activation of the caspase-9/caspase-3/GSDME signaling pathway.

### TRI-03 inhibits TrxR1 activity in melanoma cells

**FIGURE 3:**
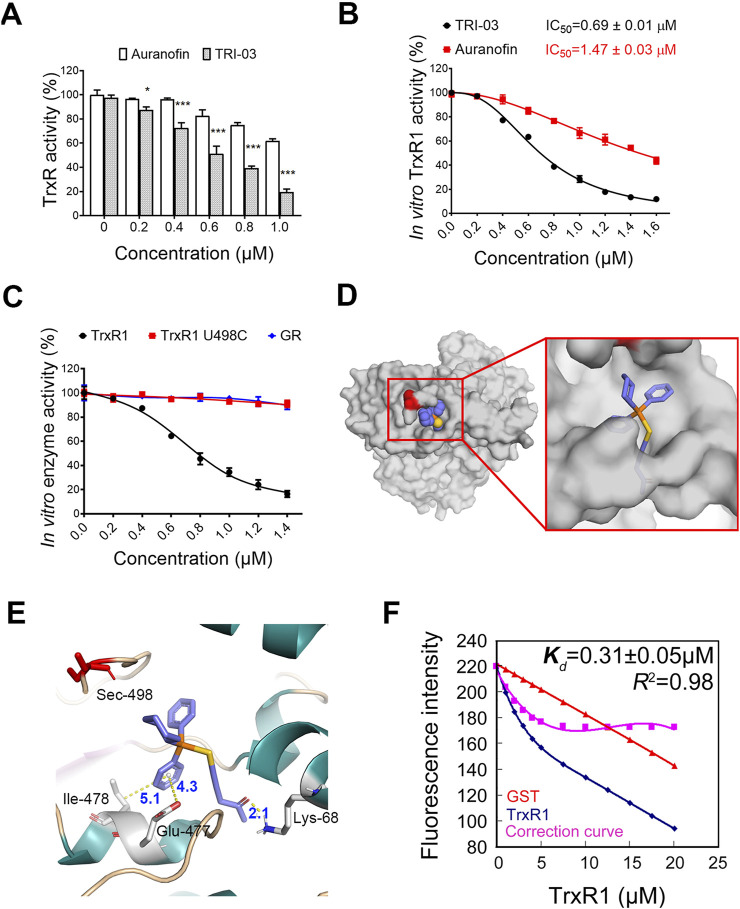
TRI-03 suppresses TrxR1 activity in melanoma cells. **(A)** After a 3-h treatment with TRI-03 or auranofin at 37°C, the total TrxR activity was measured in A375 cells. Auranofin was used as a positive control. **(B)** Purified recombinant TrxR1 protein was incubated with TRI-03 or auranofin for 2 h at different concentrations, and the activity was measured using DTNB assay. The IC_50_ values for both TRI-03 and auranofin were provided. **(C)** Purified proteins of recombinant TrxR1, TrxR1 mutant U498C, and NADPH-reduced GR were separately incubated with TRI-03 at the indicated concentrations for 2 h, and their activities were measured using the DTNB assay. **(D)** TRI-03 was docked into the binding site of TrxR1 (PDB ID: 3EAN) (overall view). **(E)** A detailed view of the binding site of TrxR1 (PDB ID: 3EAN) with TRI-03. TRI-03 was depicted using blue-colored sticks, and hydrogen, pi-anion and pi-alkyl bonds were shown as yellow dotted lines. Key residues in TrxR1 were highlighted in green or red. **(F)** The *Kd* value for the interaction between TRI-03 and TrxR1 was determined by fluorescence quenching. In this assay, TrxR1 was incubated with increasing concentrations of TRI-03, and GST protein was included as an internal filter control to account for any nonspecific effects. The fluorescence intensity was recorded as TRI-03 was sequentially added to the protein solution. A correction curve was generated to normalize the data, and the *Kd* value was calculated based on the binding curve. The Kd value is presented as the mean ± SEM, and *R* ^*2*^ represents the coefficient of determination. Most data were presented as the mean ± SD, but the enzyme activities were expressed as mean IC_50_ ± SE. ***P < 0.001, *P < 0.05, ns: not significant.

The TrxR family, serving as a key intracellular target for gold(I) complexes, features a selenocysteine residue at its catalytic site. The TrxR family includes three members: cytoplasmic TrxR1, mitochondrial TrxR2, and testis-specific TrxR3 (Bjorklund et al., 2021). TrxR1 was the most highly expressed of these isoforms (Supplementary Figure S3A). We investigated the effect of TRI-03 on TrxR1 activity in melanoma cells and found that TRI-03 administration significantly inhibited TrxR1 activity in a dose-dependent manner, whereas auranofin had only a moderate effect (Figure 3A). Further studies revealed that TRI-03 did not induce transcriptional changes or alterations in protein stability (Supplementary Figures S1B, C), confirming that this gold(I) complex specifically inhibits TrxR1 enzymatic activity in melanoma cells.

The *in vitro* inhibitory effects of TRI-03 on TrxR1 were subsequently evaluated. The IC_50_ value of TRI-03 for recombinant TrxR1 was found to be 0.69 ± 0.01 μM, substantially lower than auranofin’s IC_50_ value of 1.47 ± 0.03 μM under identical conditions (Figure 3B). Furthermore, pent-1-yn-3-one does not affect the activity of TRI-03 (Supplementary Figure S3D). We evaluated the specificity of this gold(I) complex by generating two recombinant proteins: TrxR1 U498C (where selenocysteine at position 498 is replaced with cysteine) and glutathione reductase (GR, similar in structure to TrxR1 but lacking specifically selenocysteine residues). The administration of TRI-03 resulted in a dose-dependent decrease in the enzymatic activity of wild-type TrxR1, while it had minimal effects on both TrxR1 U498C and GR (Figure 3C). These findings indicate that TRI-03 directly and selectively inhibits TrxR1, with the selenocysteine residue at position 498 being essential for this inhibition.

To determine the binding site of TRI-03 on TrxR1, we used molecular docking methods to establish a hypothetical binding mode for TRI-03 on TrxR1 (PDB ID: 3EAN) and obtained an estimated binding energy of −8.56 kcal/mol. In this model, TRI-03 interacts deeply with a pocket in TrxR1 (Figure 3D) formed by residues from two chains: Lys-68 and Glu447 in chain A and Phe-406, Leu-409, Glu-410, His-472, Cys-475, Glu-477, Ile-478, and Gln-494 in chain B. Significantly, TRI-03 forms a pi–alkyl bond (5.1 Å) with Ile-478, a pi–anion bond (4.3 Å) with Glu-477, and a hydrogen bond (2.1 Å) with Lys-68 of TrxR1 (Figure 3E). Furthermore, TRI-03 is located within 4 Å of the selenocysteine residue at the C-terminus of TrxR1 (Figure 3E). Considering the flexibility of the C-terminus (Cheng et al., 2009), especially in the reduced enzyme form, this selenocysteine residue is very likely to directly react with gold ions.

We further employed fluorescence quenching assays to confirm the interaction between TRI-03 and TrxR1 and determined the equilibrium dissociation constant (*K*_*d*_) of the TrxR1-TRI-03 complex to be 0.31 ± 0.05 μM (Figure 3F). Overall, these results indicate that this gold(I) complex directly binds to and inhibits TrxR1.

### TRI-03 stimulates intracellular ROS accumulation in melanoma cells

**FIGURE 4:**
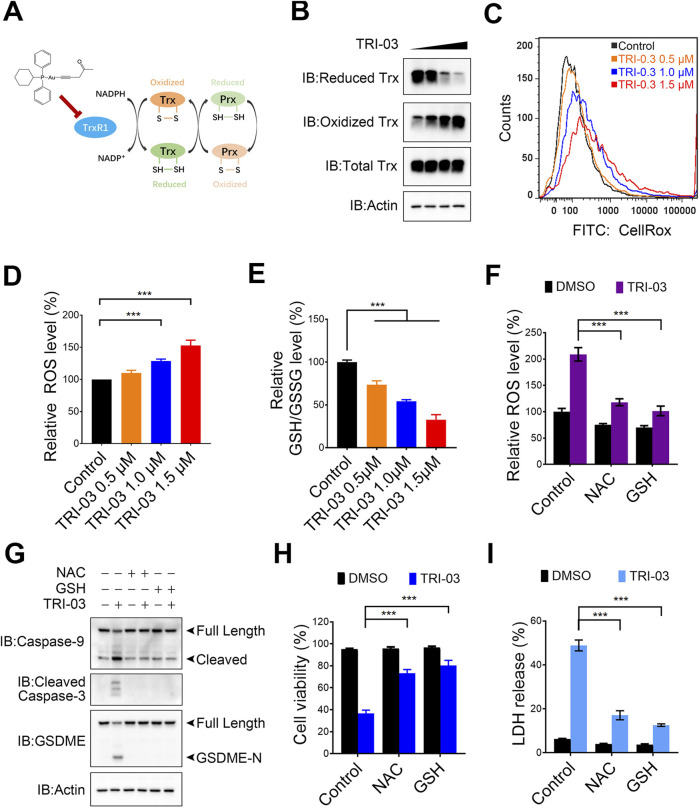
TRI-03 promotes ROS accumulation in A375 cells. **(A)** An overview of the thioredoxin system was shown. **(B)** A375 cells were treated with TRI-03 at concentrations of 0.5 μM, 1 μM, or 1.5 μM for 12 h, and the redox state of Trx was analyzed using Western blot. **(C,D)** A375 cells were exposed to TRI-03 at the specified concentrations for 12 h, stained with CellRox Green, and the cellular ROS levels were measured by flow cytometry **(C)** and quantified **(D)**. **(E)** The intracellular GSH/GSSG ratio in A375 cells was determined under the same conditions as in **(C)**. **(F)** A375 cells were pretreated with NAC (5 mM) or GSH (1 mM) for 2 h, followed by an additional 12-h incubation with TRI-03 (1 μM). ROS levels were then analyzed by flow cytometry after CellRox Green staining. **(G–I)** In another set of experiments, A375 cells were treated with NAC (5 mM) or GSH (1 mM) for 2 h before receiving TRI-03 (1 μM, 24 h) treatment. The cleavage of Caspase-3, Caspase-9 and GSDEM **(G)**, cell survival **(H)** and LDH release **(I)** were subsequently assessed. All data were presented as the mean ± SD of two or three independent experiments, ***P < 0.001.

TrxR plays an important role in converting oxidized Trx to its active form, which is essential for removing harmful intracellular ROS (Tinkov et al., 2018) (Figure 4A). A375 cells were treated with different concentrations of TRI-03 to explore how TRI-03 affects the redox state of Trx. The separation of reduced and oxidized Trx was achieved using PAO Sepharose, followed by Western blot analysis. Under normal conditions, Trx exists primarily in its reduced form. However, the ratio of oxidized to reduced Trx was significantly raised as the TRI-03 concentration increased (Figure 4B). These results suggest that TRI-03 impairs TrxR1 function, consequently weakening the efficiency of Trx reduction.

The inability of oxidized Trx to transfer electrons to Prx results in impaired H_2_O_2_ scavenging and subsequent ROS accumulation. The cellular ROS levels were measured using a fluorescence-based analysis to evaluate the effects of TRI-03. We observed dose-dependent increases in ROS levels in A375 cells treated with TRI-03 (Figures 4C,D). TRI-03 also disrupted the intracellular GSH/GSSG ratio, a key indicator of the redox status (Figure 4E). To further investigate the role of ROS in TRI-03-induced pyroptosis, A375 cells were pretreated with N-acetylcysteine (NAC) and glutathione (GSH) separately, which are antioxidants known to neutralize excessive ROS (Figure 4F). Both of these antioxidants significantly reduced TRI-03-induced cleavage of caspase-3, caspase-9 and GSDME, cell death and LDH release (Figures 4G–I). Overall, these results suggest that TRI-03 effectively induces melanoma pyroptosis, which depends on intracellular ROS accumulation.

### TRI-03 facilitates melanoma cell pyroptosis by degrading XIAP

**FIGURE 5:**
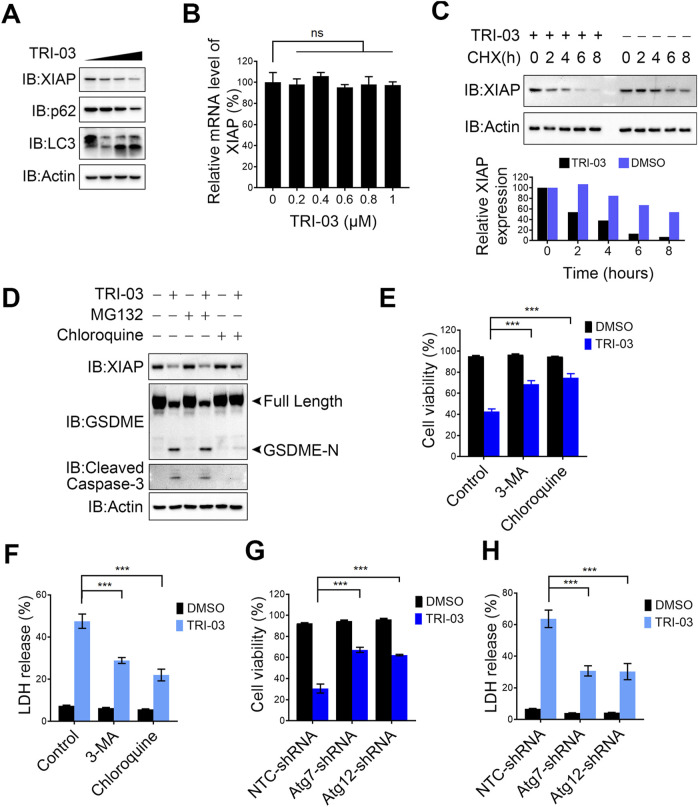
TRI-03 induces pyroptosis in melanoma cells by degrading XIAP. **(A)** A375 cells were treated with TRI-03 at concentrations of 0.5 μM, 1 μM, or 1.5 μM for 24 h, and the protein level of XIAP, LC3 and p62 were analyzed by Western blot. **(B)** A375 cells were treated with TRI-03 at various concentrations for 6 h, and the mRNA level of XIAP was analyzed by qPCR. **(C)** A375 cells were treated with CHX (100 μg/mL), and TRI-03 (1 μM) or vehicle (DMSO) for the indicated time points. XIAP protein level was examined by Western blot (upper panel), and the quantification of protein bands was analyzed using Image Lab software (lower panel). **(D)** A375 cells were cultured in the absence or presence of MG132 (10 μM) or chloroquine (20 μM) for 2 h, followed by treatment with or without TRI-03 (1 μM) for another 24 h. Subsequently, the protein levels of XIAP, GSDME and cleaved caspase-3 were analyzed by Western blot. **(E,F)** A375 cells were pretreated separately with autophagy inhibitors: 3-MA (20 μM) and chloroquine (20 μM) for 2 h before incubation with TRI-03 (1 μM) for 24 h. Cell survival **(E)** and LDH release **(F)** were determined. **(G,H)** In A375 cells, Atg7 or Atg12 were separately knocked down using the corresponding shRNA, followed by treatment with DMSO or TRI-03 (1 μM) for 24 h. Cell survival **(G)** and LDH release **(H)** were determined. All data were presented as the mean ± SD of two or three independent experiments, ***P < 0.001, ns: not significant.

We previously discovered that pent-1-yn-3-one, an alkynyl-containing compound, effectively reduces XIAP expression in melanoma cells (Supplementary Figure S1A). As a derivative of this natural compound, TRI-03 also contains an alkynyl group and a ketone moiety in the R2 unit. We observed that the administration of TRI-03 significantly decreased XIAP protein levels in a dose-dependent manner in melanoma cells (Figure 5A), as well as the conversion of LC3-I to LC3-II (Figure 5A), which is an indicator of autophagy activation. Quantitative real-time PCR confirmed that TRI-03 does not affect the mRNA level of XIAP (Figure 5B). To determine the mechanism by which TRI-03 regulates XIAP expression, we used cycloheximide (CHX) to block protein translation and found that TRI-03 clearly reduced the stability of the XIAP protein rather than through transcriptional regulation (Figure 5C). The ubiquitin‒proteasome and autophagy systems are the two main quality control pathways responsible for cellular homeostasis (Pohl and Dikic, 2019). We pretreated A375 cells with the proteasome inhibitor MG-132 and the autophagy inhibitor chloroquine. Blocking the autophagy pathway significantly inhibited the TRI-03-induced degradation of XIAP, leading to a reduction in caspase-3 activation and the cleavage of GSDME. However, blocking the proteasome pathway failed to exert this inhibitory effect (Figure 5D).

We pretreated A375 melanoma cells with chloroquine and 3-MA, another autophagy inhibitor, to further investigate the role of autophagy in TRI-03-induced cell death. We discovered that both compounds partially suppressed cell death and LDH release in response to TRI-03 treatment in melanoma cells (Figures 5E,F), indicating that TRI-03-induced pyroptosis depends at least partially on autophagy-mediated XIAP degradation. Next, we knocked down key genes in the autophagy pathways. Consistent with the results obtained with the inhibitors, silencing the expression of Atg7 and Atg12 (Supplementary Figures S4A, B), essential components of the autophagy machinery, allowed melanoma cells to partially resist death and reduced LDH release after exposure to TRI-03 (Figures 5G,H). In summary, TRI-03 degrades XIAP via the autophagy pathway, thereby increasing the activation of caspase family members and ultimately leading to GSDME-mediated pyroptosis.

### The physiological role of TRI-03 in anti-melanoma in mouse models

**FIGURE 6:**
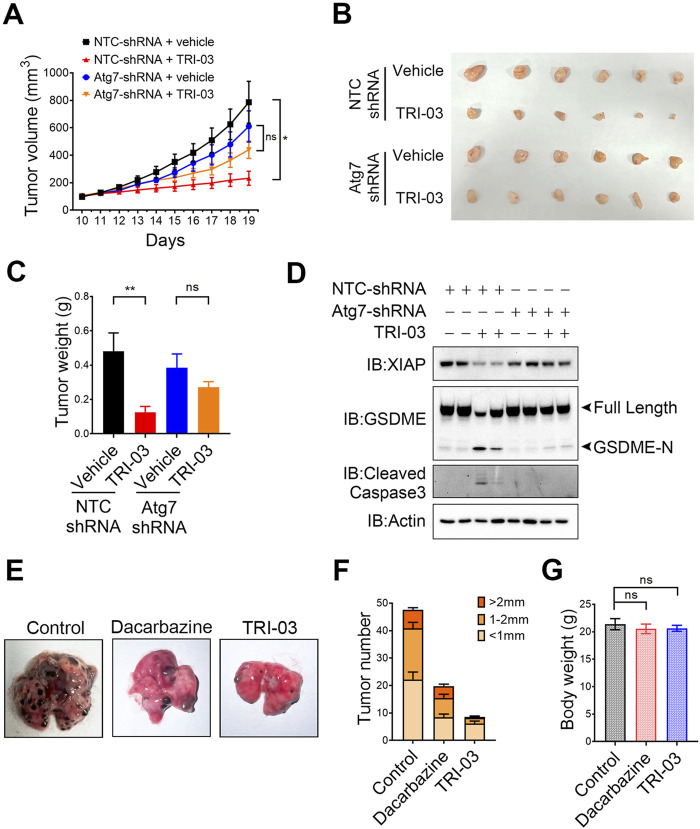
Physiological role of TRI-03 in suppressing melanoma growth in mouse models. **(A–D)** A375 cells transfected with NTC-shRNA or Atg7-shRNA were separately injected into nude mice to form subcutaneous xenografts (n = 6). Vehicle or TRI-03 (10 mg/kg) was intratumorally injected every 2 days. **(A)** Tumor growth curves in mice receiving DMSO or TRI-03 treatment schedule. **(B)** Picture of A375 xenograft. **(C)** Tumor weights. **(D)** XIAP, GSDME and cleaved caspase-3 in tumors were detected by Western blot. Actin was used to determine the amount of loading proteins. **(E–G)** B16 cells were injected into C57BL6/J mice, which were then randomly divided into three groups (n = 5). Each group received vehicle, dacarbazine (10 mg/kg), or TRI-03 (10 mg/kg) three times a week for 2 weeks. At the end of the test, lungs were harvested for imaging **(E)** and counting **(F)**. **(G)** Body weights of the mice were measured. **P < 0.01, *P < 0.05, ns: not significant.

We established melanoma xenograft models in nude mice to evaluate the role of autophagy-mediated XIAP degradation in inhibiting melanoma growth upon TRI-03 stimulation *in vivo*. A375 NTC-shRNA and A375 Atg7-shRNA cells were subcutaneously injected into the right flanks of the mice, which were then randomly divided into four groups: 1) the vehicle-treated NTC-shRNA group, 2) the TRI-03-treated NTC-shRNA group, 3) the vehicle-treated Atg7-shRNA group, and 4) the TRI-03-treated Atg7-shRNA group. Compared with the vehicle, TRI-03 significantly inhibited the growth of NTC-shRNA melanoma over a 9-day treatment period (Figure 6A). In contrast, this inhibitory effect was markedly reduced when endogenous Atg7 expression was silenced (Figure 6A).

Mice treated with TRI-03 presented a significant reduction in tumor weight, with a tumor growth inhibition rate of 74.0% (P < 0.01), However, TRI-03 had no significant inhibitory effect in the Atg7-shRNA group (P = 0.66) (Figures 6B,C). Furthermore, Western blot analysis of the NTC-shRNA group revealed that TRI-03 treatment led to decreased levels of XIAP in melanoma tissue, as well as increased cleavage of GSDME and caspase-3 (Figure 6D). However, TRI-03 failed to produce these effects in mice injected with Atg7-shRNA-transfected melanoma cells. These findings indicate that the activation of GSDME-mediated pyroptosis and the inhibition of melanoma proliferation *in vivo* at least partly depend on autophagy-mediated XIAP degradation.

Since melanoma is known for its high metastatic potential, we used another mouse model to investigate the physiological role of TRI-03 in its antimetastatic activities. B16 cells were injected into the tail vein of C57BL/6 mice, allowing the cells to migrate to the lungs and form tumors. Compared with the dacarbazine injection, the intravenous injection of TRI-03 notably reduced the number of melanoma nodules in the lungs, with a more pronounced effect (Figures 6E,F). Additionally, no obvious side effects were observed in the TRI-03-treated group. Specifically, no significant changes in body weight (Figure 6G), liver function parameters (aspartate aminotransferase (AST) and alanine aminotransferase (ALT) levels) (Supplementary Figures S5A, B), or renal function parameters (serum creatinine and blood urea nitrogen (BUN) levels) were detected (Supplementary Figures S5C, D).

Collectively, our results suggest that TRI-03 successfully inhibits melanoma growth and metastasis *in vivo* without inducing serious adverse effects and demonstrates better efficacy compared with existing clinical drugs.

## Discussion

**FIGURE 7:**
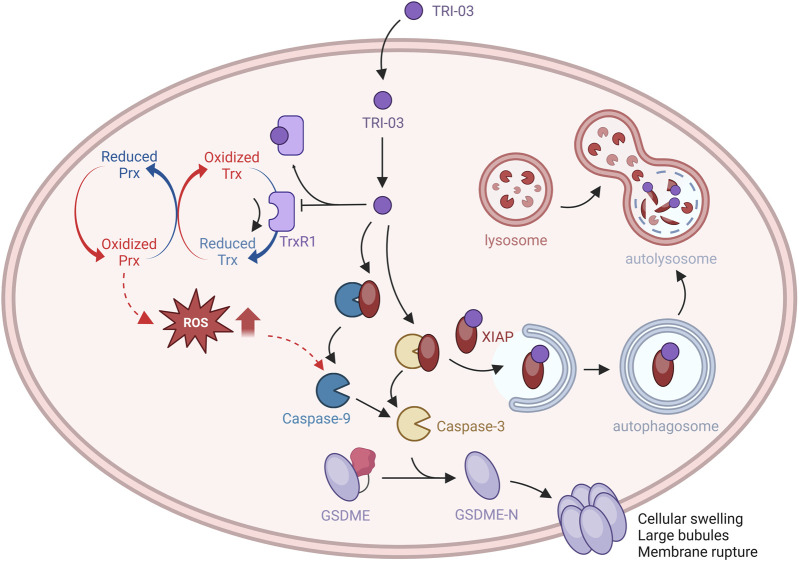
The effects of TRI-03 on Trx/TrxR-modulated pyroptosis signaling pathways.

Melanoma, the most aggressive form of skin cancer, is characterized by multiple gene mutations that render it resistant to apoptosis, posing a significant challenge for successful treatment (Davis et al., 2018). Consequently, identifying alternative therapeutic strategies is crucial. Pyroptosis, a regulated form of programmed cell death, is characterized by lytic cell death mediated by the gasdermin family (Zhu et al., 2024). In this study, we designed and synthesized a series of gold(I) complexes containing diphenyl-2-cyclohexylphosphine (R1) and alkyne (R2) units. Among these, TRI-03 directly inhibits TrxR1 activity, significantly increasing the ratio of oxidized Trx1 to reduced Trx1 and leading to increased intracellular ROS accumulation. Additionally, TRI-03 reduces XIAP expression via the autophagy pathway. This dual inhibition of TrxR1 and XIAP in melanoma cells induces substantial caspase-9/caspase-3 activation, leading to irreversible GSDME-mediated pyroptosis (Figure 7). Therefore, our research identified TRI-03 as a promising lead compound capable of overcoming melanoma resistance to apoptosis, suggesting a new approach for the development of melanoma treatments.

High expression of TrxR1 is frequently observed in malignant melanoma and plays a crucial role in maintaining the cellular redox balance and key signaling pathways that drive tumor growth, survival, and metastasis (Barral et al., 2000; Wang et al., 2014). In this study, we focused on the ability of TRI-03 to inhibit TrxR1 in melanoma cells. TRI-03 more effectively inhibited TrxR1 activity in a dose-dependent manner than did the first-generation gold complex auranofin. Molecular docking results indicated that TRI-03 directly and strongly interacts with a pocket in TrxR1, significantly affecting the selenocysteine residue (U498) at the C-terminus of TrxR1. Recent reports have highlighted the strong affinity of gold for sulfur and selenium, leading to rapid reactions with activated cysteine or selenocysteine residues to form gold–thiolates or gold–selenolates (Lu et al., 2022). The unique mechanism of action of gold compounds not only offers potential benefits in disrupting the cellular redox balance, resulting in melanoma-specific cytotoxicity, but also provides insights for the development of novel metal-based cancer treatments.

GSDME-mediated pyroptosis is a type of programmed cell death characterized by the caspase-3-dependent cleavage of GSDME, which results in the formation of large pores in the plasma membrane, cell lysis, and the release of inflammatory cytokines (Jiang et al., 2020). Pyroptosis has the potential to specifically benefit cancer therapy by triggering programmed cell death at the tumor site (Hsu et al., 2021; Christgen et al., 2022). Unlike traditional apoptosis and necrosis, pyroptosis is more effective in inducing the release of intracellular pro-inflammatory factors, such as IL-1β and IL-18, thereby promoting a robust inflammatory response and reducing tumor resistance (Li et al., 2021). However, to date, there is a limited number of studies on pyroptosis-inducing agents that effectively combat tumors. This scarcity of research makes it difficult to assess whether these pyroptosis-inducing drugs may cause severe side effects in normal tissues (Baguley, 2010). Consequently, the development of new methods to induce pyroptosis in the context of tumor therapy remains an area that requires further exploration.

Recent studies have made progress in understanding the positive regulators of GSDME-mediated pyroptosis, but the negative regulators are still largely unknown. In this study, we identified XIAP as a novel negative regulator of pyroptosis. XIAP, which was previously shown to inhibit apoptosis by binding to activated caspase-3 and caspase-9, was found to have a new function in mediating TRI-03-induced cellular pyroptosis. Elevated XIAP levels are frequently associated with a poor prognosis for patients with various cancers, including melanoma (Tu and Costa, 2020; Daoud et al., 2022). Consequently, understanding the signaling pathways involved in the function of XIAP during pyroptosis can provide insights into its role in antimelanoma effects and its potential clinical applications.

At the translational level, XIAP expression is regulated primarily by an IRES-mediated mechanism that specifically induces XIAP expression during cellular stress, such as chemotherapy drug treatment (Gu et al., 2016). However, the regulation of XIAP at the posttranslational level is not well understood. In this study, we found that TRI-03 degrades XIAP through the autophagy pathway, thereby increasing the activation of caspase family members. Autophagy can be nonselective, such as bulk autophagy, or selective, targeting specific components such as damaged organelles and aggregated or specific proteins, which play crucial roles in cellular quality control (Vargas et al., 2023). Therefore, understanding how TRI-03 regulates XIAP degradation via the autophagy-dependent pathway is of significant interest. Recent advances have improved our understanding of selective autophagy mechanisms, including the identification of autophagy receptors such as p62, NDP52, NBR1, and OPTN, which bind both cargo and ubiquitin to initiate autophagy (Johansen and Lamark, 2020). Consequently, future research on autophagy-mediated XIAP degradation may not only identify and validate key molecules and signaling pathways involved in selective autophagy but also facilitate the development of new drugs in melanoma therapy and interventions to enhance autophagy capabilities.

## Data Availability

The raw data supporting the conclusions of this article will be made available by the authors, without undue reservation.
