# Supplementary material for: A dual inhibitor of TrxR1 and XIAP induces pyroptosis in melanoma
Source: Front Cell Dev Biol. 2025 May 23;13:1542356. doi: 10.3389/fcell.2025.1542356 (PMC12141336; doi:10.3389/fcell.2025.1542356)
Supplement: Supplementary file 1 [file DataSheet1.docx]

**A DUAL INHIBITOR OF TrxR1 AND XIAP INDUCES PYROPTOSIS IN MELANOMA**

**Yuan Wang^1,2#*^, Xiangmei Li^1*^, Xinyue Dong^1*^, Haokun Yuan^3^, Ruiqin Fang^4^, Ran Zhang^5^, and Wei-jia Wang^6,7#^**

**SUPPLEMENTARY INFORMATION**

**Supplementary Figure 1**


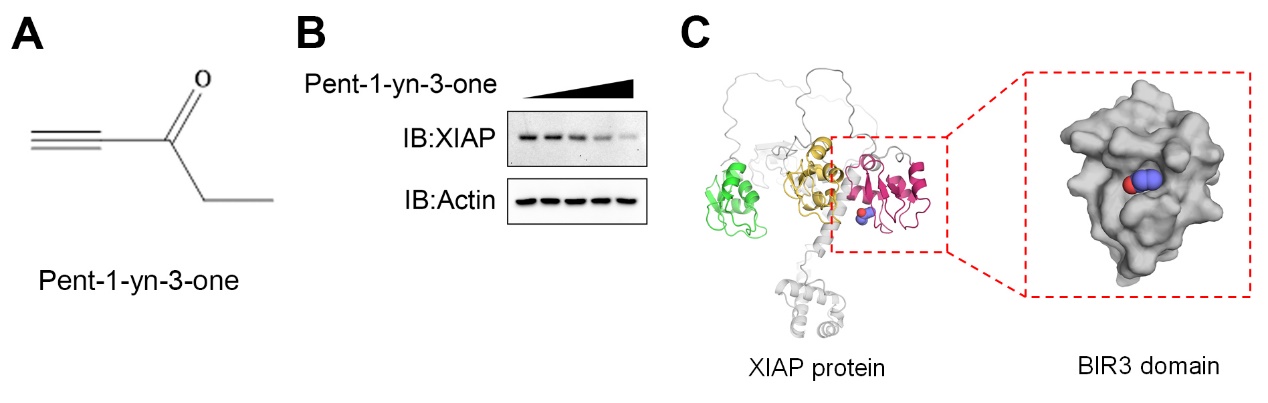


**Supplementary Figure 1**. Pent-1-yn-3-one decreases XIAP expression in melanoma cells. A) The structure of pent-1-yn-3-one. B) A375 cells were treated with Pent-1-yn-3-one at concentrations of 0.5 μM, 1 μM, 2 μM and 5 μM for 24 hours, and the protein level of XIAP was analyzed using western blot. C) Pent-1-yn-3-one was docked into the BIR3 domain of XIAP.

**Supplementary Figure 2**


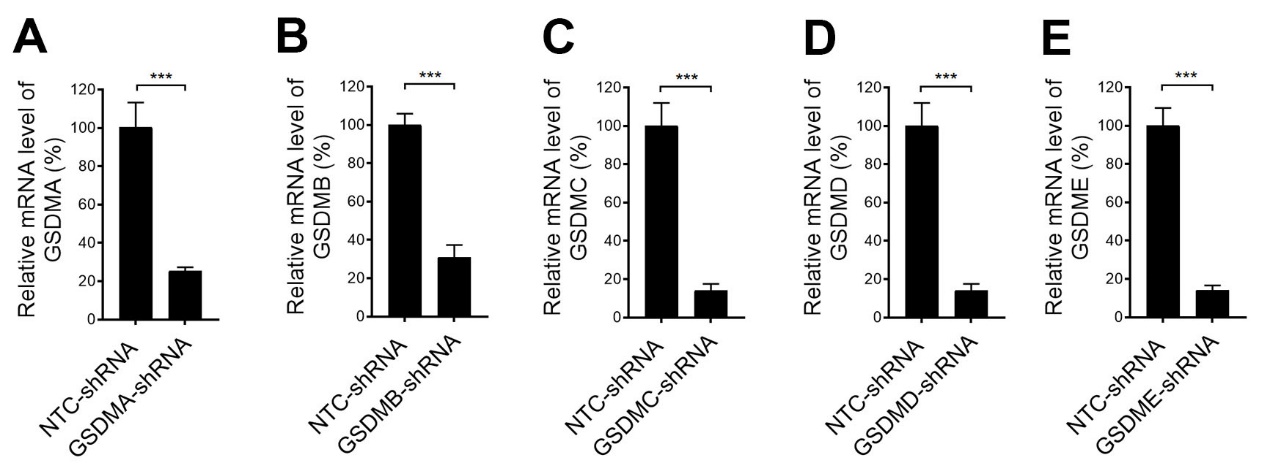


**Supplementary Figure 2**. The efficiency of the knockdown was evaluated. A-E) The expression of endogenous GSDMA (A), GSDMB (B), GSDMC (C), GSDMD (D) and GSDME (E) was separately knocked down by corresponding shRNAs in A375 cells. All data are presented as the mean ± SD of three independent experiments. ***P < 0.001.

**Supplementary Figure 3**


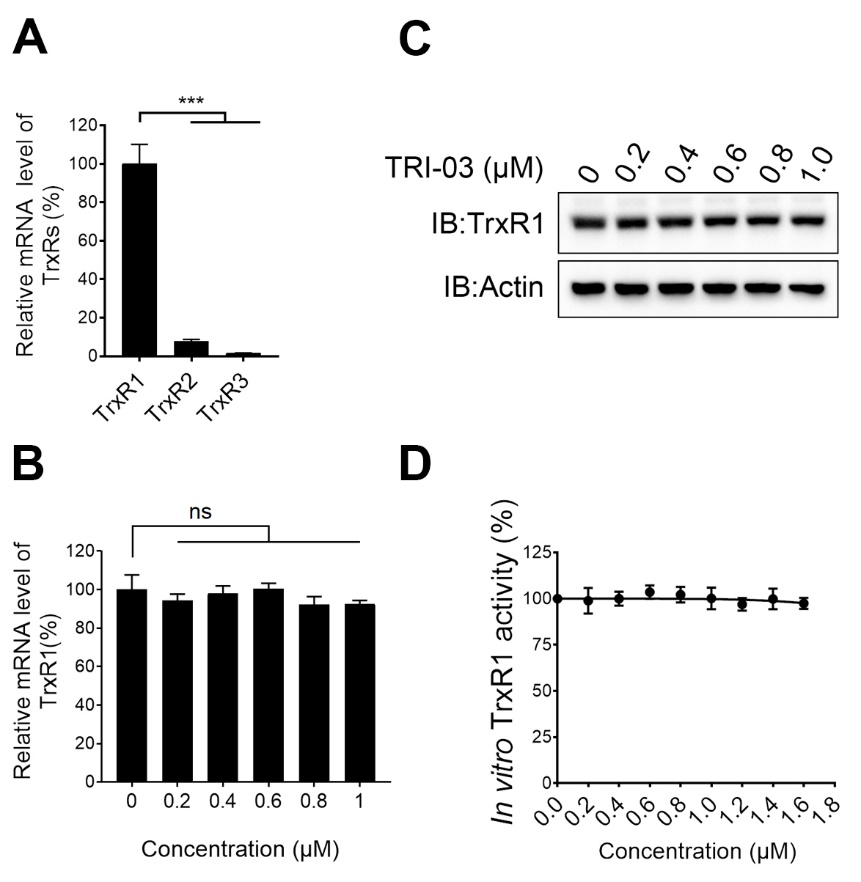


**Supplementary Figure 3.** The effect of TRI-03 on TrxRs. A) The mRNA levels of TrxR1, TrxR2 and TrxR3 in A375 cells were detected by qPCR. B) In A375 cells, the mRNA level of TrxR1 was assessed by qPCR after treatment with increasing concentrations of TRI-03. C) In A375 cells, the protein level of TrxR1 was assessed by western blot after treatment with increasing concentrations of TRI-03. D) Purified recombinant TrxR1 protein was incubated with pent-1-yn-3-one for 2 hours at different concentrations, and the activity was measured using DTNB assay. All data are presented as the mean ± SD. ***P < 0.001, ns: not significant.

**Supplementary Figure 4**


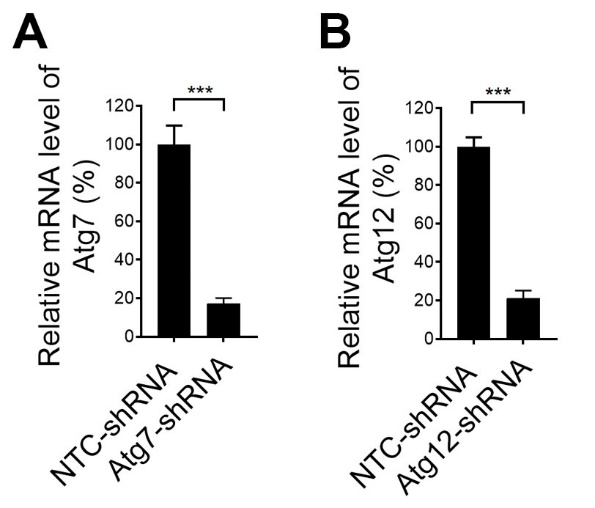


**Supplementary Figure 4.** The efficiency of the knockdown was evaluated. A-B) The expression of endogenous Atg7 (A) and Atg12 (B) was separately knocked down by corresponding shRNAs in A375 cells. All data are presented as the mean ± SD of three independent experiments. ***P < 0.001.

**Supplementary Figure 5**


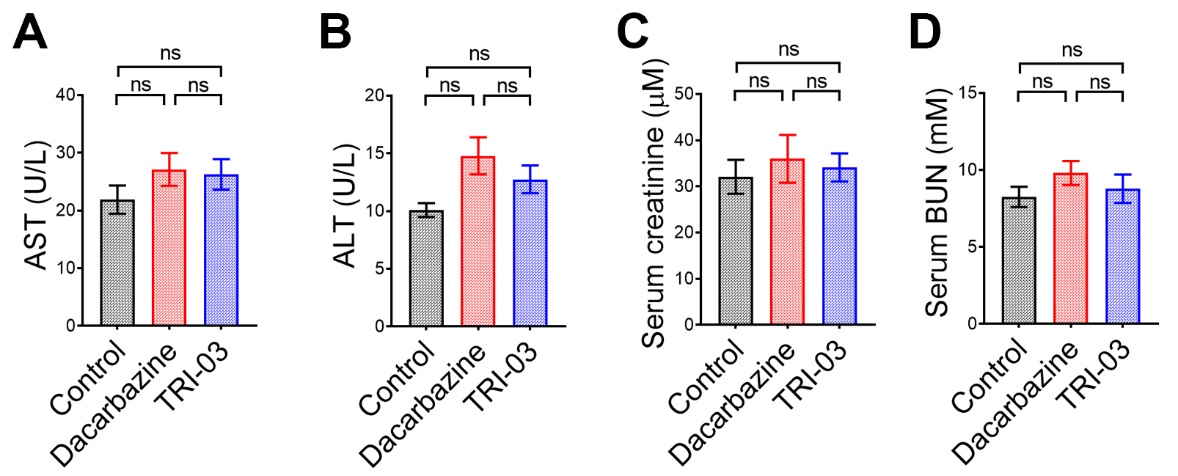


**Supplementary Figure 5.** Biochemical parameters in mice model. A-D) At the endpoint of the experiment, peripheral blood serum was collected from the mice for biochemical analysis. A) Serum aspartate aminotransferase (AST) level. B) Serum alanine aminotransferase (ALT) level. C) Serum creatinine level. D) Blood urea nitrogen (BUN) level. ns: not significant.
